# How to Evaluate a Regional Telemedical Care Network Focusing on Critically Ill Children? Results from the Consensus-Based Development of an Evaluation Design

**DOI:** 10.3390/children12030313

**Published:** 2025-02-28

**Authors:** Josephine Mathiebe, Gabriele Müller, Jochen Schmitt, Sebastian Brenner, Stefan Winkler, Anne Schawohl, Franziska Waurig, Madlen Scheibe

**Affiliations:** 1 Center for Evidence-Based Healthcare, Faculty of Medicine and University Hospital Carl Gustav Carus, TUD Dresden University of Technology, Fetscherstraße 74, 01307 Dresden, Germanymadlen.scheibe@ukdd.de (M.S.); 2 Department of Pediatrics, Faculty of Medicine and University Hospital Carl Gustav Carus, TUD Dresden University of Technology, Fetscherstraße 74, 01307 Dresden, Germany

**Keywords:** pediatric emergency medicine, intensive care units, telemedicine, pediatric telehealth, health care quality, access, and evaluation, regional care network, primary care, rural regions, telemedicine innovations

## Abstract

**Background:** The medical care of critically ill children requires special expertise in pediatric emergency and intensive care medicine. In Germany, this expertise is mainly available in specialized centers at maximum-care hospitals due to the small number of cases of critically ill children. Telemedical care networks, such as the Pediatric-Tele-Intensive-Care Network Saxony, offer a solution for networking these centers with regional hospitals providing basic or standard care. However, the evaluation of these networks represents a major challenge, especially because it involves a heterogeneous target group of patients with various diseases. This article reports on the evaluation design development process for such a network, under equal participation of all relevant stakeholders, and presents the resulting evaluation design. **Methods:** The methods used were a literature review, two workshops, and a survey of all relevant stakeholders (representatives of 17 partner clinics, 4 external experts in pediatric intensive care, and 2 patient representatives). **Results:** The evaluation design contained proposals for a care objective, outcomes, and the corresponding databases (project database, clinic database, and survey database) to investigate these. **Conclusions:** Our study can offer other researchers and stakeholders a methodological approach and template for their evaluation design. It is the first published, consented evaluation design for a telemedical care network in pediatric intensive care. When developing and realizing an evaluation design for (tele)medical interventions, including the perspectives of relevant stakeholders, from the outset, it is essential to achieve a high level of commitment to the implementation of the evaluation.

## 1. Introduction

The medical care of critically ill children requires special expertise in pediatric emergency and intensive care medicine to reduce complication rates and the risk of mortality, thereby improving the long-term prognosis. In Germany, due to the low number of cases of critically ill children, this expertise is mainly available in specialized centers at maximum-care hospitals. Therefore, hospitals providing basic or standard care are confronted with various healthcare challenges. The treatment of critically ill children requires an interdisciplinary perspective and cooperation between several specialist disciplines, typically not available 24/7 in-house at hospitals providing basic or standard care. Currently, these hospitals receive support from specialized centers mainly via telephone calls. These calls often take place without a standardized procedure, and the medical staff in the specialized centers have no opportunity to audio-visually assess the patient’s state of health. Additionally, no specialized pediatric critical care transport teams are available as standard medical care in Germany. Furthermore, every transport represents a risk for the patient, and avoiding transfer to continue treatment in a hospital close to home may be advantageous.

To address these challenges, many studies have investigated telemedicine solutions in intensive care. For example, meta-analyses by Wilcox et al. and Chen et al. showed a reduction in intensive care unit (ICU) mortality and hospital mortality, in general, through the use of telemedicine for critically ill patients [1,2]. A significant reduction in mortality among critical care patients through telemedicine has also been found in other international studies and systematic reviews [3,4,5,6,7,8,9]. Additionally, due to earlier and more consistent treatment provided by tele-ICU, complication rates, mortality, and length of stay in the ICU and hospital have been significantly reduced [10,11].

Evidence on telemedicine in intensive care for critically ill children is still comparatively limited concerning the practical implementation and the quality of care. For example, Ghbeis et al. and Munoz et al. described the results of a process evaluation of the use of telemedicine in the intensive care treatment of children [12,13]. Improvements in the quality of care of sick children are particularly noticeable in intensive care because this patient group has the lowest reserves to tolerate deviations from optimal care. At the same time, they benefit from even small improvements in the quality of care [14]. However, some hospitals providing basic or standard care do not have ICUs, which further limits the evidence on the implementation of telemedicine in this field.

**Figure 1:**
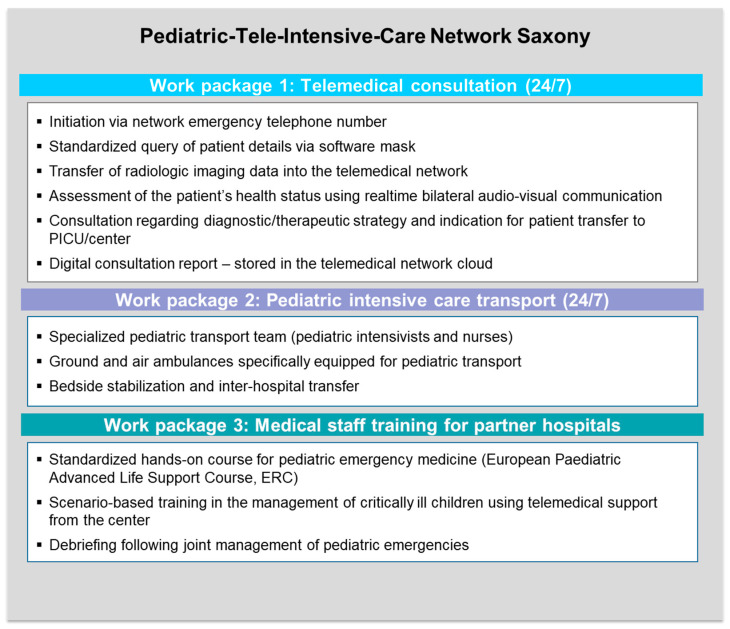
Pediatric-Tele-Intensive-Care Network Saxony. Description of work packages. PICU, pediatric intensive care unit; ERC, European Resuscitation Council.

To optimize the healthcare of critically ill children in hospitals providing basic or standard care in the German State of Saxony, the Pediatric-Tele-Intensive-Care Network Saxony project was initiated in 2022. The goal of the project is to improve the quality of care and outcomes of critically ill children in these hospitals by establishing a pediatric tele-intensive care network. The region of Eastern Saxony in Germany was selected as an example. For this purpose, the Clinic and Polyclinic for Pediatrics and Adolescent Medicine at Dresden University Hospital (hereafter “the center”) joined forces with 17 other regional partner clinics for an innovative model project. The quality of care is intended to be improved with the implementation of three work packages, shown in Figure 1.

The study population consisted of all critically ill children between the ages of the 28th day of life and the end of the 18th year of life who were treated in one of the 17 regional partner clinics during the project period, required support in diagnosis and treatment from the center, and for whom the services of the Pediatric-Tele-Intensive-Care Network Saxony were used during the project period. Secondary study populations were the relatives of the included patients and the medical and nursing staff who used or offered the telemedical care network services.

Telemedical networks for pediatric intensive care are also reported internationally. For example, studies from the USA report that not every hospital has a pediatric intensive care unit or pediatric critical care physicians available in-house. As part of the networks, there is also the opportunity to use audio-visual communication to receive support for the medical care of critically ill children from a pediatric critical care physician. The reported results include reduced severity of symptoms among critically ill children who were transferred with telemedical support compared to those without, as well as improved triage for appropriate patient care in comparison to telephone calls [15,16]. In contrast to these networks, the Pediatric-Tele-Intensive-Care Network Saxony includes an additional work package to provide standardized training for medical staff. To evaluate the Pediatric-Tele-Intensive-Care Network Saxony, an evaluation design including defined primary and secondary outcomes and a valid data basis were needed. The evaluation findings provide the scientific basis for health policy decisions, especially on the extent to which the structures established as part of the project can be consolidated as permanent healthcare structures.

The development of the evaluation design for such a network posed several challenges. Firstly, the three work packages formed the framework within suitable outcomes, and measurement instruments had to be selected. Secondly, we had to consider that the study population is a heterogeneous patient group with heterogeneous diseases and correspondingly low case numbers, both in total and for individual diseases. Thirdly, the chosen care goals and quality indicators had to be relevant for the evaluation of the network, but, at the same time, their measurement and documentation had to be feasible for all participating clinics. Fourthly, a controlled study design for the evaluation was not an option due to the aforementioned heterogeneous diseases in this patient group and correspondingly low case numbers. Hence, the use of frequently used outcomes, such as mortality or morbidity, was not feasible.

Given the reported challenges, we aimed to develop a consensus-based evaluation design with all stakeholders involved (physicians and nursing staff of the partner clinics and the center, external experts in pediatric intensive care, and patient representatives). This article aims to report on the development process of the objective, outcomes, and data sources for the evaluation of a regional telemedical care network, with equal participation of the involved stakeholders, and the finally consented evaluation design.

## 2. Materials and Methods

### 2.1. Literature Review

The theoretical basis of the draft evaluation design was a literature review. It included scientific publications and medical guidelines focusing on the effects of using telemedicine in intensive medical care (for critically ill children) or suggesting outcomes, outcome measurement instruments, or quality indicators for measuring these effects [1,2,3,4,5,6,7,8,9,10,11,12,13,14,17,18,19,20]. Following the literature research and outlined preliminary considerations, we developed a draft evaluation design that contained proposals for a care objective, research questions, outcomes, and quality indicators, and the corresponding data basis and outcome measurement instruments to investigate these.

### 2.2. Workshops and Survey

In the second step, we conducted two workshops and a survey with representatives of all regional partner clinics participating in the Pediatric-Tele-Intensive-Care Network Saxony, external experts in pediatric intensive care, and patient representatives (parents of formerly critically ill children). These aimed to discuss and modify the draft evaluation design, resulting in a final evaluation design.

#### 2.2.1. Selection of Participants

When selecting the workshop and survey participants, we ensured that professional and scientific expertise in the subject area, as well as the perspectives of those affected, were represented. Such a group composition is also common in the development of medical guidelines in Germany by the Association of the Scientific Medical Societies in Germany [21]. A balanced composition of workshop participants created the right conditions for the critical assessment of the draft evaluation design and the comprehensive identification of possible practical problems in its implementation. This also avoided possible bias due to the influence of particular interests [21].

The following participants were invited to our workshops and survey:

- representatives from the medical and nursing staff of the 17 participating regional partner clinics;
- four external experts in pediatric emergency and intensive care medicine;
- two patient representatives (parents of children who had received pediatric emergency or intensive care treatment);
- five representatives from the medical and nursing staff of the center;
- two representatives of the research institute evaluating the project.

#### 2.2.2. First Workshop

The first workshop took place face-to-face in July 2022 at the Dresden University Hospital. It aimed to introduce the draft evaluation design to the workshop participants and obtain their interdisciplinary feedback.

The participants were invited to ask any questions for understanding, comment on the evaluation design, and suggest further research questions, outcomes and quality indicators, and data basis and outcome measurement instruments relevant for a comprehensive evaluation. All feedback was recorded and subsequently incorporated into a revised version of the evaluation design. Altogether, 12/17 clinics, 4/4 external experts, and 2/2 patient representatives (parents of formerly critically ill children) participated in the first workshop.

#### 2.2.3. Survey

To gather structured feedback from all representatives on the revised evaluation design, a survey was conducted in August 2022. In the questionnaire, all parts of the evaluation design were prepared in a structured manner. The participants rated their agreement with the presented care objective, research questions, outcomes and quality indicators, and data basis and outcome measurement instruments. They indicated whether they felt that specific changes to the evaluation design were necessary, e.g., that formulations should be modified or new outcomes or data should be added to the evaluation design. The willingness of the 17 regional partner clinics to contribute to the following tasks was also queried:

■ Informing the children treated within the network and their relatives about Pediatric-Tele-Intensive-Care Network Saxony and its evaluation, obtaining their consent for their data to be passed on to the evaluating institution;
■ Distributing questionnaires to the medical staff of their clinics and relatives of critically ill children as a data source for the evaluation;
■ Providing information on included children from their clinical database to the evaluating institution at the end of the project.

The doctors and nursing staff at the regional partner clinics were asked to complete only one questionnaire for each clinic. The survey was not conducted anonymously because the feedback provided the basis for the second workshop; therefore, knowing who gave which feedback was important. The participants were informed of this in advance and asked for their consent. Consequently, 13/17 clinics, 3/4 external experts, and 2/2 patient representatives (parents of formerly critically ill children) participated in the survey.

#### 2.2.4. Second Workshop

**Figure 2:**
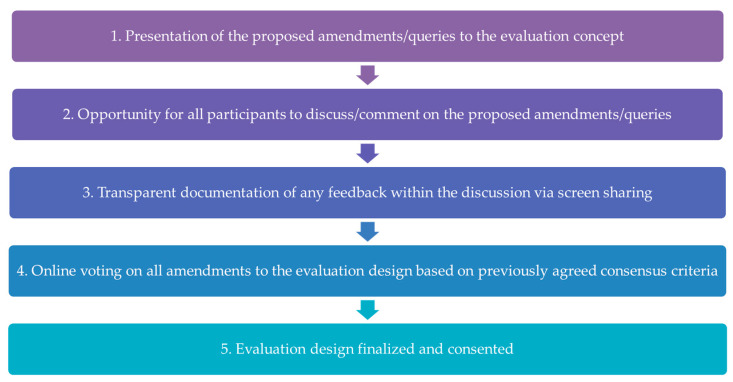
Steps in the structured consensus-building workshop.

Based on the survey results, a second workshop was conducted online in September 2022. This structured consensus-building workshop aimed to discuss the feedback and proposed changes to the evaluation design from the survey, vote on proposed changes, and finalize the evaluation design. The rules for structured consensus-building defined by the Association of the Scientific Medical Societies in Germany were followed [22] by conducting a structured, transparent, and equal consensus-building procedure. All participants agreed that 75% approval was required for a suggested amendment to be adopted. If less than 75% agreed, the suggested amendment was not adopted. Each partner clinic had one vote. Representatives of the center and the evaluating institute were not entitled to vote. Figure 2 shows the consensus-building steps.

Altogether, 9/17 clinics, 3/4 external experts, and 0/2 patient representatives participated in the second workshop, reflecting that it was particularly attended by representatives who had suggested amendments. The non-participating representatives were excused from attending the second workshop for resource reasons (time constraints). However, they were involved before and after the second workshop, for example, by receiving the agreed evaluation design. Thereby, they had the opportunity to comment on it at any time, and they finally agreed on the evaluation design. Altogether, 36 responses were discussed in the second workshop. A total of 19 proposed amendments were voted on, and the rest were queries that could be clarified during the joint discussion. Of the 19 proposed amendments, 14 were adopted, with at least 75% in favor.

## 3. Results

### 3.1. Study Design

We chose a study design based on an exploratory approach without a control group. Therefore, we were not testing hypotheses but investigating primary and secondary research questions. Furthermore, we carried out a complete survey of all included patients. Thus, no sample size estimation or power calculation was conducted. Instead, the focus of the study was a comprehensive collection and analysis of the available data to obtain initial results and formulate hypotheses.

### 3.2. Objective and Research Questions

**Figure 3:**
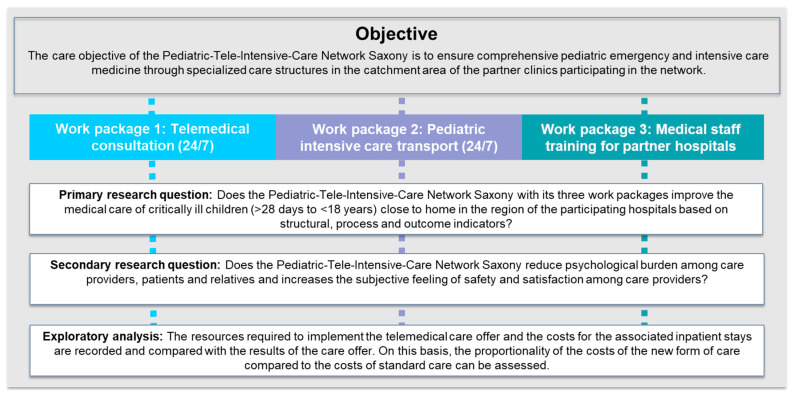
Overview of the agreed care objective, research questions, and exploratory analysis for work packages of the Pediatric-Tele-Intensive-Care Network Saxony.

For the final evaluation design, the workshop participants agreed on a common care objective of the Pediatric-Tele-Intensive-Care Network Saxony and formulated primary and secondary research questions. Additionally, an exploratory analysis was agreed on. Figure 3 provides an overview of the agreed care objective, the research questions, and the exploratory analysis for the three work packages of the Pediatric-Tele-Intensive-Care Network Saxony.

### 3.3. Primary Research Question and Outcomes

The workshop participants agreed that the primary research question should investigate whether the Pediatric-Tele-Intensive-Care Network Saxony, with its three work packages, improves the medical care of critically ill children close to home. In Germany, a critically ill child is usually transported to the nearest suitable hospital. Regional hospitals providing basic or standard care can then request support from the Pediatric-Tele-Intensive-Care Network Saxony. Table 1 shows the primary outcomes for the description of care (primary research question) for all three work packages, including the respective data sources and the project partners responsible for documentation or data collection. The secondary outcomes for the description of care (primary research question) for all three work packages are listed in Table 2.

### 3.4. Secondary Research Question and Outcomes

The workshop participants agreed that the evaluation should investigate whether the Pediatric-Tele-Intensive-Care Network Saxony reduces psychological burden among medical and nursing staff, patients, and relatives and increases satisfaction among medical and nursing staff (secondary research question). Table 3 lists the secondary outcomes for the investigation of the secondary research question for all three work packages, including the respective data sources and the project partners responsible for documentation or data collection. As shown in Table 2, we are also investigating the proportion of children with emergency teleconsultations who did not have to be transferred to the center due to telemedical support. Therefore, the questionnaire for the relatives also includes items that explore their perspective on the possibility of continued treatment in a hospital close to home with telemedical support.

### 3.5. Exploratory Analysis and Outcomes

The workshop participants decided that the resources required to implement the telemedical care network should also be examined to assess the proportionality of the costs of this new form of care compared to standard care (exploratory analysis). The aim was to assess the feasibility of implementing the Pediatric-Tele-Intensive-Care Network Saxony for standard care. Table 4 shows the outcomes of the evaluation of resources that were agreed on in the workshops.

### 3.6. Study Population Description

To describe the study population as part of the evaluation, the documentation of patient information was also specified in the workshops (Table 5).

### 3.7. Data Sources

**Figure 4:**
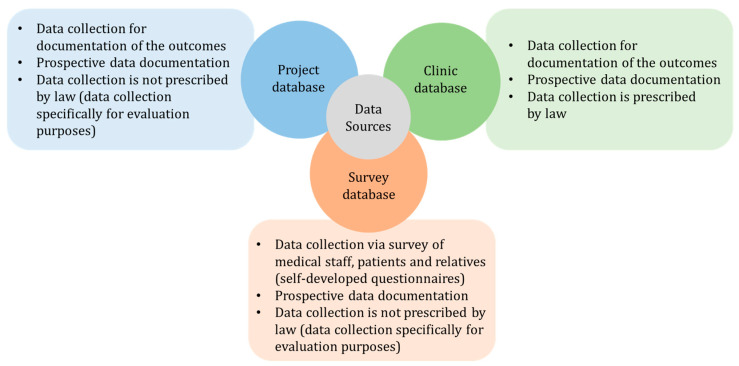
Overview of the three agreed databases of the Pediatric-Tele-Intensive-Care Network Saxony for analyzing the defined primary and secondary outcomes.

The workshop participants agreed that three data sources should be used for the evaluation: a project data base, a clinic data base, and a survey data base. These three data sources are described in Figure 4.

As described in Figure 4, German clinics are required by law to collect certain data. Selected items from these datasets were used for the evaluation. These items and outcomes are listed in Table 2 and Table 4 as the data source “clinic database.” All other data required for the analysis of the defined outcomes were additionally collected as part of the project and documented in a project database and a survey database. These outcomes are listed in Table 1, Table 2, Table 3 and Table 4 as the data sources “project database” and “survey database.” In the workshops, the documentation of the outcomes presented in Table 1, Table 2, Table 3 and Table 4 was considered feasible by all participants. The use of the data from the databases presented in Figure 4 for the evaluation required the informed consent of legal guardians.

## 4. Discussion

Evidence of the use of telemedicine in the intensive care of critically ill children is limited. The evaluation of the Pediatric-Tele-Intensive-Care Network Saxony can provide new findings for this field of research. To the authors’ knowledge, no current evaluation design exists for regional pediatric tele-intensive care networks. To close this research gap, the project team developed a specific evaluation design. This was undertaken in close dialogue with all stakeholders involved: representatives of all participating clinics, external experts in pediatric intensive care, and patient representatives.

The first step in developing the evaluation design presented here was to agree on the objective for the evaluation of the telemedicine care network with the stakeholders involved (Figure 3). At the international level, the World Health Organization Regional Office for Europe aims to “Set national goals and targets related to health in Member States” [23] (p. 16). Accordingly, the German Network for Health Services Research calls for the consensus of measurable healthcare targets by the stakeholders involved to “ensure effective, affordable and high-quality health care” [24] (p. 972). For our evaluation, the joint agreement on the objective for evaluating the Pediatric-Tele-Intensive-Care Network Saxony was thus important to be able to derive appropriate research questions, outcomes, quality indicators, measurement instruments, and documentation processes to evaluate the achievement of this objective. The results of the evaluation will then serve as a basis for the evidence-based further development of the telemedical care network, especially for health policy decisions on the extent to which the structures established as part of the project can be consolidated as permanent healthcare structures. In Germany, there are currently structural challenges in pediatric inpatient care (e.g., closures of pediatric departments and nursing staff shortages) [25,26,27]. Therefore, funding will be provided during the project period to determine if the Pediatric-Tele-Intensive-Care Network Saxony, with its three modules, can help to cope with these structural challenges. Furthermore, this project aims to determine the conditions under which the network can be transferred to other regions and how reimbursement models for such telemedical networks for pediatric intensive care should be designed (explorative analysis).

Regarding the outcomes selected for the evaluation, the inclusion of different perspectives was also reflected. In particular, the secondary outcomes included the perspectives of medical and nursing staff, as well as patients and relatives, such as concerning their psychological stress. The analysis of these perspectives is also recommended in the context of quality development in the healthcare sector and is thus also important for the evaluation of telemedicine projects [20,28]. In addition, the results of the identified outcomes will be used to formulate initial hypotheses, so that these can be compared with the findings of other studies. For example, Dharmar et al. report that the “overall quality of care” was assessed higher by two coders in cases where telemedical consultations were conducted compared to those with telephone calls or without consultations [29,30]. Furthermore, there are indications that the use of telemedical consultations reduces the number of patient transfers between hospitals [31]. With regard to long-term outcomes such as mortality and complications in pediatric intensive care, the evidence is still limited [31,32]. We cannot make any statements about such outcomes due to our study design. At the same time, O’Brien et al. report evidence for increasing the availability of special expertise in pediatric intensive care by the use of telemedicine [32]. These findings highlight the importance of this study to examine whether the Pediatric-Tele-Intensive-Care Network Saxony can optimize structural challenges in Germany [25,26,27].

Furthermore, using data that are already documented in routine care is an advantage for the evaluation of telemedicine projects [20]. As the results show, we used such data in the “clinic database” data source. However, it became clear during the consensus-building that many of the agreed outcomes are not documented in routine care and must be collected additionally for evaluation using the “project database” data source. This shows that the evaluation of such telemedical care networks for their transfer to standard care requires a certain amount of effort, which, in turn, demands a high level of commitment from the partner clinics. At the same time, the joint determination of the responsibilities for documenting the outcomes offers the potential to further increase the commitment of the involved stakeholders.

The focus of our study was a process evaluation. To measure the effect of such newly established healthcare structures, it is advantageous to increase the intervention and measurement duration to increase the case numbers of the study population. Therefore, the healthcare structures already implemented here must be not only used temporarily but continued in the long term and used as a starting point for further large-scale intervention studies. Further studies with a longer duration and different designs (e.g., a stepped wedge design) are necessary to be able to measure further outcomes with a focus on effective evaluation to complement our presented outcomes.

### Strengths and Limitations

One strength of our study is that it is the first published, consensus-based evaluation design for a telemedical care network with many partner clinics in pediatric intensive care. Our study, presenting the process of consenting to an objective, outcomes, and data sources for the evaluation of a telemedical network, can offer other researchers and stakeholders a methodological approach and template for their evaluation design.

Our evaluation design was developed to evaluate a regional pediatric tele-intensive-care network consisting of three work packages: telemedical consultation (24/7), pediatric intensive care transport (24/7), and medical staff training for partner hospitals. For other telemedicine networks, the entire design can be used as a template, but selecting only individual parts for their evaluation design is also possible, e.g., if only telemedical consultations were conducted.

Another strength of our study is its development of an evaluation design for a very heterogeneous patient group, involving the perspectives of medical and nursing staff, researchers, and patients and relatives. We found that the joint discussion of outcomes and the examination of the feasibility of the documentation increased the commitment of the partner clinics in the network. This is reflected by 12/17 clinics, 4/4 external experts, and 2/2 patient representatives who participated in the first workshop, as well as in the 13/17 clinics, 3/4 external experts, and 2/2 patient representatives who participated in the survey.

Our study also has limitations. The Pediatric-Tele-Intensive-Care Network Saxony was evaluated until December 2024, and the data analysis will be finished by June 2025. We thus cannot yet make any conclusion on the extent to which the selected databases or documented data were appropriate for measuring the selected outcomes. Furthermore, since this study did not use a controlled design, this limits our conclusions on the effectiveness of the network (e.g., on the mortality and morbidity of critically ill children).

Regarding the feasibility of the secondary outcomes, the survey with patients could not be conducted as planned. As the patient group includes critically ill children, some were not conscious during the emergency and were not aware of the telemedical application. Additionally, patients need time to recover after intensive medical treatment and are thus not in a healthy state to take part in a survey. At the same time, surveying patients and relatives soon after treatment was important to minimize recall bias. Furthermore, we used questionnaires to investigate the perspectives of medical staff and relatives. Therefore, we cannot exclude the possibility of a bias due to social desirability in the response behavior. To minimize potential bias, the medical staff and relatives sent their questionnaires anonymously by mail to the evaluating institution. The medical staff were surveyed at two measuring points. Possible social desirability would probably occur at both measuring points. Further studies that link the results of the questionnaires to other data sources (e.g., by linking the data to clinical outcomes, such as severity of illness) would be useful to conduct subgroup analyses for a more differentiated understanding and to validate the findings. When using additional data collection methods, we also recommend taking into account the potential psychological burden on families and the limited resources of medical staff.

## 5. Conclusions

To our knowledge, our study is the first published, consented evaluation design for a telemedical care network in pediatric intensive care. It can be used for other evaluation projects, even if it may need to be adapted to the specific framework conditions of the setting under investigation. In general, when developing and realizing an evaluation design for (tele)medical interventions, including the perspectives of medical staff, patients, external experts, and the evaluating research institutions from the outset is essential. This may be the best way to achieve a high level of commitment to the implementation of the evaluation. It becomes even more important when less required data are routinely collected, and more data must be collected specifically for evaluation purposes.

## Figures and Tables

**Table 1:** Primary outcomes for work packages 1 to 3 for the description of care (primary research question).

Outcome	Data Source	Responsibility for Documentation
Work package 1: Telemedical consultation (24/7)		
Proportion of children for whom a physician was connected telemedically <15 min after an emergency call	Project database	Center
Proportion of children with re-consultation with previous appointment	Project database	Center
Work package 2: Pediatric intensive care transport (24/7)		
Proportion of transported critically ill children whose condition remained stable during intensive care transport (recorded before transport and on arrival at the center, based on list of indicators)	Project database	Center
Work package 3: Medical staff training for partner hospitals		
Proportion of regional partner clinics with the specified minimum number of employees trained in the European Paediatric Advanced Life Support (EPALS) or American Heart Association Pediatric Advanced Life Support (AHA PALS) courses with a valid certificate at the beginning and end of the project	Project database	Regional partner clinics

**Table 2:** Secondary outcomes for work packages 1 to 3 for the description of care (primary research question).

Outcome	Data Source	Responsibility for Documentation
Work package 1: Telemedical consultation (24/7) Work package 2: Pediatric intensive care transport (24/7)		
Mean duration of inpatient stay per child (total duration of inpatient stay)	Clinic database	Center; regional partner clinics
Mean duration of stay in the intensive care unit per child	Clinic database	Center; regional partner clinics
Work package 1: Telemedical consultation (24/7)		
Number of patients included in work package 1	Project database	Center
Number of emergency teleconsultations	Project database	Center
Mean time from realization of the emergency to the first audiovisual contact with the pediatrician connected via telemedicine	Project database	Center
Mean time from phone call to first audiovisual contact with the pediatrician via telemedicine	Project database	Center
Mean Pediatric Logistic Organ Dysfunction-2 (PELOD-2) score during emergency teleconsultations	Project database	Center
Proportion of children with emergency teleconsultations who had to be transferred to the center despite telemedical support	Project database	Center
Proportion of children with emergency teleconsultations who did not have to be transferred to the center due to telemedical support	Project database	Center
Total number of teleconsultations	Project database	Center
Mean frequency of teleconsultations per patient	Project database	Center
Reason for teleconsultation	Project database	Center
Proportion of pediatric emergencies among the reasons for teleconsultation (including documentation of priority reasons for consultation)	Project database	Center
Frequency with which the diagnosis was amended via telemedicine	Project database	Center
Frequency with which therapy was adjusted or extended via telemedicine	Project database	Center
Frequency with which procedures were guided via telemedicine	Project database	Center
Proportion of critically ill children receiving telemedical care who were finally discharged from the hospital close to home (including interim transfer to the center)	Clinic database	Regional partner clinics
Work package 2: Pediatric intensive care transport (24/7)		
Number of patients included in work package 2	Project database	Center
Mean time between alert and departure of intensive care transport from the center	Project database	Center
Proportion of intensive care transports for which departure from the center took place within 60 min after the alarm was raised	Project database	Center
Proportion of high-quality intensive care transport based on predefined quality indicators	Project database	Center
Proportion of patients in work package 2 who were not previously in work package 1	Project database	Center
Rate of interhospital transfers of patients From hospital close to home to the center From the center to hospital close to home Further transfer to the center	Clinic database	Center; regional partner clinics
Mean number of interhospital transfers per patient	Clinic database	Center; regional partner clinics
Work package 3: Medical staff training for partner hospitals		
Number of European Paediatric Advanced Life Support (EPALS) training courses conducted within the project Number of trained physicians Number of trained nursing staff	Project database	Center
Proportion of regional partner clinics with a structured emergency concept after the implementation of telemedicine at the beginning and the end of the project, based on predefined quality indicators	Project database	Center based on on-site visits

**Table 3:** Secondary outcomes for work packages 1 to 3 for the description of psychological burden among medical staff, patients, and relatives and satisfaction among medical staff (secondary research question).

Outcome	Data Source	Responsibility for Documentation
Work package 1: Telemedical consultation (24/7) Work package 2: Pediatric intensive care transport (24/7) Work package 3: Medical staff training for partner hospitals		
Psychological burden, satisfaction, and subjective perception of patient safety among medical and nursing staff	Survey database (self-developed questionnaire)	Distribution of questionnaires through regional partner clinics and center
Work package 1: Telemedical consultation (24/7) Work package 2: Pediatric intensive care transport (24/7)		
Psychological burden among patients	Survey database (self-developed questionnaire)	Distribution of questionnaires through regional partner clinics and center shortly before hospital discharge
Psychological burden among relatives	Survey database (self-developed questionnaire)	Distribution of questionnaires through regional partner clinics and center shortly before hospital discharge
Work package 1: Telemedical consultation (24/7)		
Proportion of helpful teleconsultations for calling medical staff at partner clinics	Survey database (self-developed questionnaire)	Sending link to online survey to regional partner clinics by center
Proportion of teleconsultations for which the questions of medical staff at partner clinics could be answered via telemedicine	Survey database (self-developed questionnaire)	Sending link to online survey to regional partner clinics by center

**Table 4:** Secondary outcomes for work packages 1 to 3 for the description of resources required to implement telemedical care offering Pediatric-Tele-Intensive-Care Network Saxony (exploratory analysis).

Outcome	Data Source	Responsibility for Documentation
Work package 1: Telemedical consultation (24/7)		
Costs of implementing telemedical software and hardware	Project database	Company that provides telemedical software and hardware
Costs of maintenance of telemedical software and hardware	Project database	Company that provides telemedical software and hardware
Personnel costs of implementing technology in partner clinics	Project database	Company that provides telemedical software and hardware; regional partner clinics
Work and time expenditure for teleconsultations at center, classified by profession	Project database	Center
Work and time expenditure for teleconsultations at partner clinics, classified by profession	Project database	Center
Inpatient costs per child across all clinics where the child was treated, based on Diagnosis Related Group (DRG) codes and outpatient lump sums	Clinic database	Center; regional partner clinics
Work package 2: Pediatric intensive care transport (24/7)		
Work and time expenditure for intensive care transport for children at the center, classified by profession	Project database	Center
Costs of interhospital transfers and transportation of patients	Project database	Center
Number of transports avoided through the use of telemedicine	Project database	Center
Average inpatient costs per child across all clinics where the child was treated	Clinic database	Center; regional partner clinics
Work package 3: Medical staff training for partner hospitals		
Costs of EPALS training in all clinics	Project database	Center; regional partner clinics

**Table 5:** Patient information for description of study population.

Patient Information	Data Source	Responsibility for Documentation
Hospital ID	Clinic database	Regional partner clinics
Patient ID	Clinic database	Regional partner clinics
Age at time of hospital admission	Clinic database	Regional partner clinics
Gender	Clinic database	Regional partner clinics
Year at time of hospital admission	Clinic database	Regional partner clinics
Diagnoses, operations, and procedure codes for children treated by telemedicine	Clinic database	Center or regional partner clinics
PELOD-2 score of children who were included in work package 1 or 2 (lowest score within the first 24 h)	Project database	Center
Death (yes/no) of children included in work package 1 or 2	Clinic database	Center or regional partner clinics
Number of children with access to specialist expertise through implementation of telemedicine in partner clinics	Data from regional statistics	Evaluating institution

## Data Availability

The original contributions presented in the study are included in the article. Further inquiries can be directed to the corresponding author.
